# Sodium Orthovanadate Changes Fatty Acid Composition and Increased Expression of Stearoyl-Coenzyme A Desaturase in THP-1 Macrophages

**DOI:** 10.1007/s12011-019-01699-2

**Published:** 2019-03-29

**Authors:** Jan Korbecki, Izabela Gutowska, Marta Wiercioch, Agnieszka Łukomska, Maciej Tarnowski, Arleta Drozd, Katarzyna Barczak, Dariusz Chlubek, Irena Baranowska-Bosiacka

**Affiliations:** 1 Department of Biochemistry and Medical Chemistry, Pomeranian Medical University, Powstańców Wlkp. 72 Av., 70-111 Szczecin, Poland; 2 Department of Biochemistry and Human Nutrition, Pomeranian Medical University, Broniewskiego 24 Str., 71-460 Szczecin, Poland; 3 Department of Physiology, Pomeranian Medical University, Powstańców Wlkp. 72 Av., 70-111 Szczecin, Poland; 4 Department of Conservative Dentistry and Endodontics, Pomeranian Medical University, Powstańców Wlkp. 72 Av., 70-111 Szczecin, Poland

**Keywords:** Sodium orthovanadate, Fatty acids, Macrophage, THP-1, Desaturase

## Abstract

Vanadium compounds are promising antidiabetic agents. In addition to regulating glucose metabolism, they also alter lipid metabolism. Due to the clear association between diabetes and atherosclerosis, the purpose of the present study was to assess the effect of sodium orthovanadate on the amount of individual fatty acids and the expression of stearoyl-coenzyme A desaturase (SCD or Δ^9^-desaturase), Δ^5^-desaturase, and Δ^6^-desaturase in macrophages. THP-1 macrophages differentiated with phorbol 12-myristate 13-acetate (PMA) were incubated in vitro for 48 h with 1 μM or 10 μM sodium orthovanadate (Na_3_VO_4_). The estimation of fatty acid composition was performed by gas chromatography. Expressions of the genes *SCD*, *fatty acid desaturase 1* (*FADS1*), and *fatty acid desaturase 2* (*FADS2*) were tested by qRT-PCR. Sodium orthovanadate in THP-1 macrophages increased the amount of saturated fatty acids (SFA) such as palmitic acid and stearic acid, as well as monounsaturated fatty acids (MUFA)—oleic acid and palmitoleic acid. Sodium orthovanadate caused an upregulation of *SCD* expression. Sodium orthovanadate at the given concentrations did not affect the amount of polyunsaturated fatty acids (PUFA) such as linoleic acid, arachidonic acid, eicosapentaenoic acid (EPA), docosapentaenoic acid (DPA), and docosahexaenoic acid (DHA). In conclusion, sodium orthovanadate changed SFA and MUFA composition in THP-1 macrophages and increased expression of *SCD*. Sodium orthovanadate did not affect the amount of any PUFA. This was associated with a lack of influence on the expression of *FADS1* and *FADS2*.

## Introduction

Vanadium is a metal that forms numerous inorganic compounds and complexes with organic substances. They are the subject of growing interest among researchers thanks to their antitumor properties [1]. All vanadium compounds are competitive inhibitors of protein tyrosine phosphatases (PTP) [2, 3]. In experiments on cancer cells, vanadium compounds inhibited cell cycle at checkpoints G_0_/G_1_, G_1_/S, and G_2_/M [4–9]. This is partly related to the inactivation of PTP involved in the correct course of the cell cycle [10]. Vanadium compounds also act pro-apoptotically on tumor cells [4–7, 9]. In particular, they cause the opening of the mitochondrial permeability transition pore which initiates apoptosis [11]. They also increase the expression of Bax and decrease the expression of Bcl-2, i.e., proteins regulating apoptosis [9]. Vanadium compounds also inhibit the epithelial–mesenchymal transition, which inhibits the formation of tumor metastases [12]. Due to these properties, they are intensively tested for use as antineoplastic drugs [1, 13].

There are also advanced studies on the potential use of vanadium compounds in the treatment of diabetes [14, 15]. Vanadium compounds, due to the inhibition of PTP, increase the phosphorylation of proteins on tyrosine residues. This causes changes in various signaling pathways. In particular, vanadium compounds by inhibiting PTP-1B cause an increase in phosphorylation of the insulin receptor [16–19]. Thanks to this, they abolish insulin resistance and potentiate the effect of insulin. They also strengthen the signal transmission from the insulin receptor and inhibit phosphatase and tensin homolog (PTEN). PTEN is an enzyme that catalyzes a reverse reaction to that catalyzed by phosphatidylinositol 3-kinases (PI3K) [20]. Nevertheless, vanadium compounds not only increase the action of insulin but also exert other acts than insulin. They can inhibit the activity of protein kinase A (PKA) which inhibits gluconeogenesis and lipolysis [21]. Vanadium compounds, when compared with insulin, also have a more pro-mitogenic effect inter alia, by affecting the activity of mitogen-activated protein kinases (MAPK) cascades [22].

Previous studies carried out in vivo [23–25] and in vitro [26] confirm the antidiabetic and insulin-enhancing properties of vanadium compounds, in particular vanadyl sulfate (VOSO_4_), sodium orthovanadate (Na_3_VO_4_), and the organic derivatives: bis(ethylmaltolato)oxovanadium(IV) (BEOV) and bis(maltolato)oxovanadium(IV) (BMOV). These compounds reduce blood glucose levels in many ways. In the muscles, they increase the expression of GLUT4, which increases the absorption of glucose from the blood. In liver and muscle cells, vanadium compounds stimulate glycogen synthesis [27] and increase glucose processing via the glycolysis pathway [27, 28]. They also reduce the intensity of gluconeogenesis [29]. Vanadium compounds also reduce cholesterol and LDL levels, which were very elevated in streptozotocin-induced [30] or alloxan-induced [31] diabetic rats. Vanadium compounds also cause an increase in the number of beta-cells in the pancreas of streptozotocin-induced diabetic rats [24, 25]. Clinical trials involving VOSO_4_ have shown that vanadium compounds can be used in therapy [32–34]. It was shown that at a blood concentration of approximately 4 μM (75 mg VOSO_4_ daily, route of administration: oral 5 mg/day/kg body weight VOSO_4_) was not toxic, even after 6-week therapy of patients with type 2 diabetes mellitus (T2DM) [33, 34] or supplementation with insulin through 2.5 years of therapy of patients with type 1 diabetes mellitus (T1DM) [32]. In a higher dose (300 mg orally), it caused mild diarrhea and malaise [32]. However, the therapeutic window for vanadium compounds is very narrow. Vanadium compounds in a dose above 30 mg/day/kg body weight are toxic, cause oxidative stress, and are harmful to the liver and kidneys. That has been proven in experiments on broilers [35] and on rats [36] and mice [36]. Vanadium compounds accumulate in the acidic environment of mitochondria in the form of decavanadate, which disturbs the functioning of these organelles [37, 38].

Diabetes has not only increased blood glucose levels, but also increased levels of plasma lipids, such as total cholesterol, low-density lipids (LDL), and triglyceride (TAG) as demonstrated in streptozotocin-induced [30] or alloxan-induced [31] diabetic rats as well as in patients with T2DM [39]. Increased blood glucose causes oxidative stress and inflammatory reactions in the blood vessels [40]. This process, combined with an increased amount of lipids in the plasma, causes the formation of oxysterols which are accumulated in macrophages [41]. This results in the formation of foam cells in the blood vessels and inflammation, resulting in atherosclerotic lesions. This increases the prevalence of atherosclerosis in patients with T2DM [42].

Macrophages play an important role in diseases associated with diabetes, such as nephropathy or diabetic retinopathy [43–45]. Infiltration and accumulation of these cells occur in the kidney and retina, especially in diabetes. In addition, elevated glucose levels result in macrophages producing and secreting various proinflammatory cytokines and reactive oxygen species (ROS) that contribute to the development of diabetic nephropathy and retinopathy.

An important role in the course of atherosclerosis is played by the macrophages and lipid metabolism in these cells. Therefore, the main objective of the study was to investigate the effect of selected vanadium compounds on the concentration of individual fatty acids and the expression of desaturases responsible for the formation of unsaturated bonds in fatty acids in macrophages. THP-1 macrophages grown with sodium orthovanadate at 1 μM and 10 μM were used for this purpose. These are the concentrations at which the vanadium compounds exhibit hypoglycemic properties and do not show toxic properties in humans and laboratory animals [32, 34, 46–49].

## Materials and Methods

### Cell Culture

THP-1 cells are a monocyte cell line commonly used in research on inflammatory reactions and atherosclerotic mechanisms [50–54]. Cultures of THP-1 cells (ATCC, Rockville, USA) were grown at 37 °C in 5% CO_2_ on RPMI-1640 medium (BIOMED-LUBLIN, Poland) with the addition of 10% FBS (ALAB, Poland), along with penicillin (40 U/ml) and streptomycin (40 mg/l) (Sigma–Aldrich, Poland). Cells with a viability of over 97% were placed into 6-well plates, 3 × 10^6^ wells altogether. The number of cells and their viability were determined using a Bright Line Hemacytometer (Sigma–Aldrich, Poznań, Poland) and trypan blue staining [55]. After inoculation, THP-1 monocytes were differentiated into macrophages by adding 100 nM phorbol 12-myristate 13-acetate (PMA) (carrier: DMSO) (Sigma–Aldrich, Poland) to the culture. After 24 h of incubation, the cells were washed with PBS (BIOMED-LUBLIN, Poland) and incubated in Na_3_VO_4_ (Sigma–Aldrich, Poland) (carrier: PBS). One micrometer and 10 μM Na_3_VO_4_ were used in the experiment. These concentrations were determined on the basis of in vitro studies on the antidiabetic properties [32, 34, 49, 56] and antineoplastic properties [4–7] of vanadium compounds. Cells were incubated in a medium supplemented with FBS. After 48 h of incubation with Na_3_VO_4_, THP-1 macrophages were scraped from the plate. After centrifugation (4 °C, 800×*g*, 10 min), the supernatant was discarded and the obtained cell pellet was frozen at − 80 °C for further analysis.

### Isolation and Analysis of Fatty Acid Concentration

The fatty acids from the collected cells were extracted using Folch mixture [57] (2:1, chloroform:methanol), and heneicosanoic acid (21:0) was added as an internal standard to the collected cells. The fatty acids were saponified and methylated with KOH and BF_3_ in methanol. Extraction of the obtained fatty acid methyl esters was then carried out with hexane. They were then analyzed by gas chromatography, with the use of an Agilent Technologies 7890A GC System (SUPELCOWAX™ 10 Capillary GC Column (15 m × 0.1 mm × 0.1 μm)) (Supelco, Bellefonte, PA, USA). The following chromatographic conditions were applied: from an initial temperature of 60 °C increasing at a rate of 40 °C/min to 160 °C, then increasing at a rate of 30 °C/min to 190 °C, and then increasing at a rate of 30 °C/min to 230 °C for 2.6 min, where it was maintained for 4.9 min. The total analysis took approximately 8 min. The gas flow rate was 0.8 ml/min; the carrier gas was comprised of hydrogen. The identification of fatty acids was done by comparing their retention times with those of commercially available standards. The fatty acid concentrations were determined based on standard curves and were expressed in mg/ml.

### Quantitative Real-time Polymerase Chain Reaction

Quantitative analyses of mRNA expression of stearoyl-coenzyme A desaturase (*SCD*), fatty acid desaturase 1 (*FADS1*), and fatty acid desaturase 2 (*FADS2*) were performed by two-step reverse transcription PCR. Total RNA was extracted from cells using an RNeasy Kit (Qiagen, USA). cDNA was prepared from 1 μg of total cellular RNA in 20 μl of reaction volume using a FirstStrand cDNA synthesis kit and oligo-dT primers (Fermentas, USA). The quantitative assessment of mRNA levels was performed by real-time RT-PCR using an ABI 7500Fast instrument with Power SYBR Green PCR Master Mix reagent. Real-time conditions were as follows: 95 °C (15 s), 40 cycles at 95 °C (15 s), and 60 °C (1 min). According to melting point analysis, only one PCR product was amplified under these conditions. Each sample was analyzed in two technical replicates, and the mean *C*_t_ values were used for further analysis. The relative quantity of the target, normalized to the endogenous control *GAPDH* gene and relative to a calibrator, is expressed as 2^−∆∆Ct^ (fold difference), where *C*_t_ is the threshold cycle, ∆*C*_t_ = (*C*_t_ of target genes) − (*C*_t_ of endogenous control gene), and ∆∆*C*_t_ = (∆*C*_t_ of samples for target gene) − (∆*C*_t_ of calibrator for the target gene). The following primer pairs were used: *FADS1* forward: CCAACTGCTTCCGCAAAGAC, *FADS1* reverse: GCTGGTGGTTGTACGGCATA, *FADS2* forward: TGACCGCAAGGTTTACAACAT, *FADS2* reverse: AGGCATCCGTTGCATCTTCTC, *SCD* forward: TTCCTACCTGCAAGTTCTACACC, *SCD* reverse: CCGAGCTTTGTAAGAGCGGT.

### Determination of Protein Content in the Sample

The results of the fatty acid content in the cells were converted to the protein content on the sample, which was determined using a Micro BCA Protein Assay Kit (Thermo Scientific, Pierce Biotechnology, USA) and spectrophotometer (UVM340, ASYS). The determination used biscynchonia acid (BCA), which allows detection of Cu^1+^ copper ions formed during Cu^2+^ reduction by proteins in alkaline environment. As a result of the chelation reaction of two molecules of BCA acid with one Cu^1+^ copper ion, the sample becomes violet. The method is based on the measurement of absorbance of the test substance at a wavelength of 562 nm. There is a linear relationship between the increase in protein concentration and intensity of the color.

### Statistical Analysis

The obtained results were analyzed using the Statistica 10.0 software package. The arithmetical mean ± SD was calculated for each of the studied parameters. The distribution of results for individual variables was obtained with the Shapiro–Wilk *W* test. As most of the distributions deviated from the normal distribution, non-parametric tests were used for further analyses. To assess the differences between the groups studied, the non-parametric Kruskal–Wallis ANOVA followed by the Mann–Whitney *U* test was used. A probability at *p* ≤ 0.05 was considered statistically significant.

## Results

### Sodium Orthovanadate Increased the Amount of Saturated Fatty Acids in THP-1 Macrophages

**Fig. 1:**
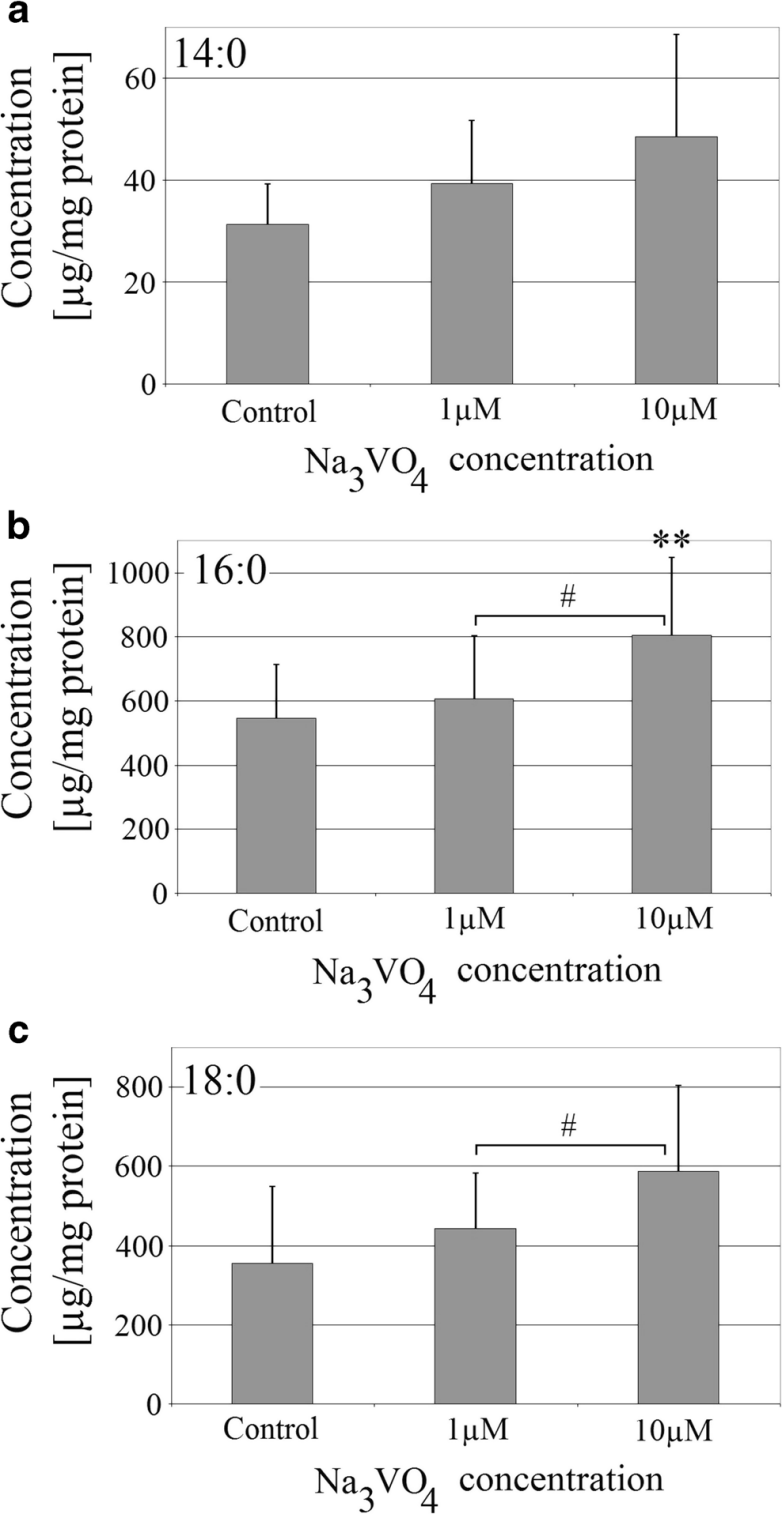
Effect of sodium orthovanadate on SFA concentration in THP-1 macrophages. The effect of sodium orthovanadate on the amount of **a** myristic acid, **b** palmitic acid, and **c** stearic acid. PMA-activated macrophages of the THP-1 cell line were cultured at two concentrations of sodium orthovanadate. After 48 h of incubation, the cells were scraped and analyzed using a gas chromatograph. Data represent means ± SD for six independent experiments. Double asterisks indicate statistically significant difference in the amount of fatty acid in macrophages relative to control with PBS, *p* ≤ 0.01. A number sign indicates a statistically significant difference in the amount of fatty acid in macrophages between two concentrations of sodium orthovanadate, *p* ≤ 0.05

Sodium orthovanadate in THP-1 macrophages increased the amount of saturated fatty acids (SFA) (Fig. 1). At 10 μM, it statistically significantly increased the amount of palmitic acid by almost 50% (*p* = 0.005). The vanadium compound tested increased statistically the amount of palmitic acid and stearic acid in comparison with the two tested concentrations (*p* = 0.041 and *p* = 0.032 appropriately).

### Sodium Orthovanadate Increased the Amount of Monounsaturated Fatty Acids in THP-1 Macrophages

**Fig. 2:**
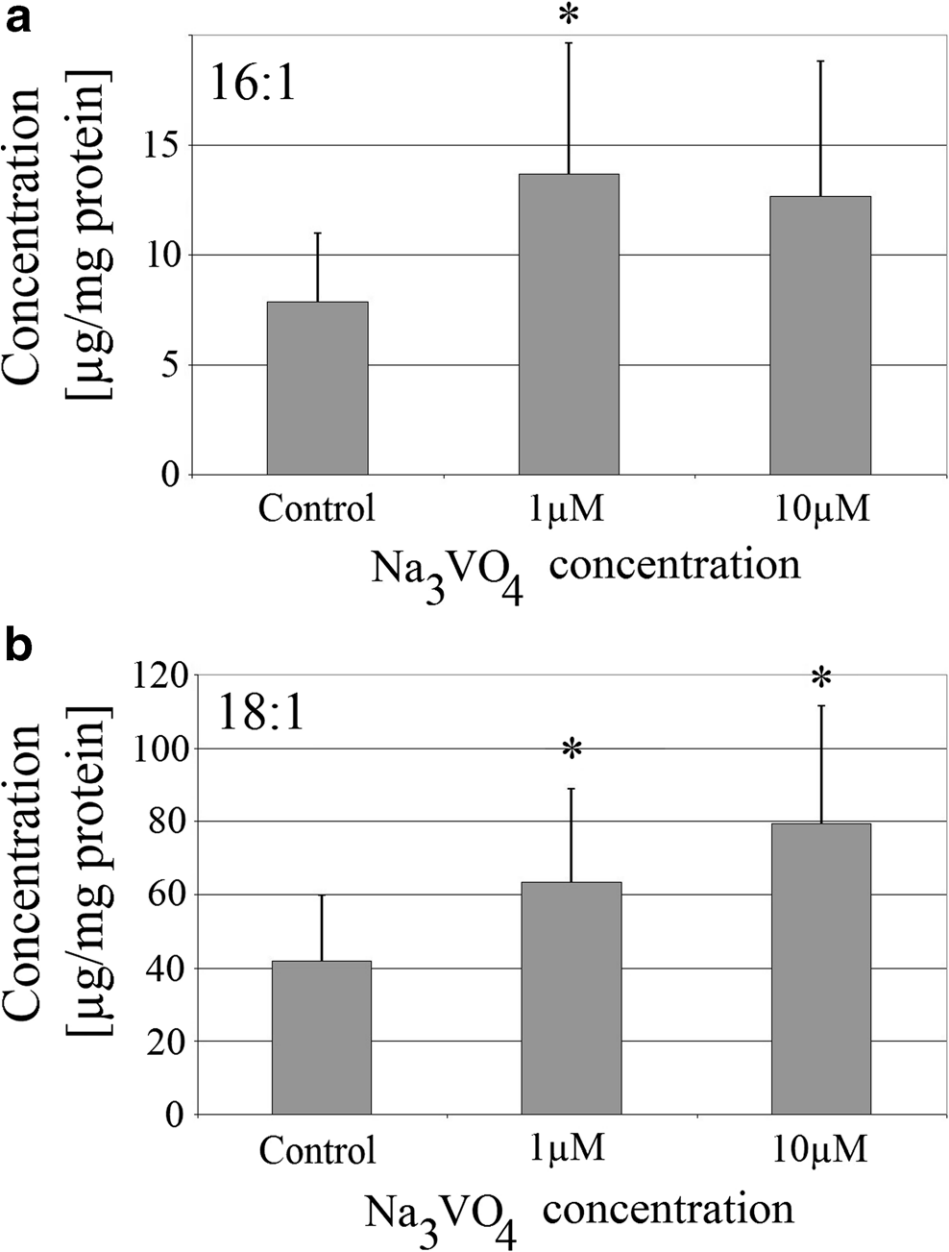
Effect of sodium orthovanadate on the concentration of MUFA in THP-1 macrophages. The effect of sodium orthovanadate on the amount of **a** palmitoleic acid and **b** oleic acid. PMA-activated macrophages of the THP-1 cell line were cultured at two concentrations of sodium orthovanadate. After 48 h of incubation, the cells were scraped and analyzed using a gas chromatograph. Data represent means ± SD for six independent experiments. An asterisk indicates a statistically significant difference in the amount of fatty acid in macrophages relative to control with PBS, *p* ≤ 0.05

Sodium orthovanadate increased the amount of monounsaturated fatty acids (MUFA) in THP-1 macrophages (Fig. 2). It statistically significantly increased the amount of oleic acid at both concentrations tested. At a concentration of 1 μM, the concentration of this fatty acid increased by 50% (*p* = 0.032), and with a concentration of 10 μM by 90% (*p* = 0.012). At the 1 μM concentration, the tested vanadium compound significantly increased the amount of palmitoleic acid by 70% (*p* = 0.036).

### Sodium Orthovanadate Did Not Affect the Amount of Polyunsaturated Fatty Acids in THP-1 Macrophages

**Fig. 3:**
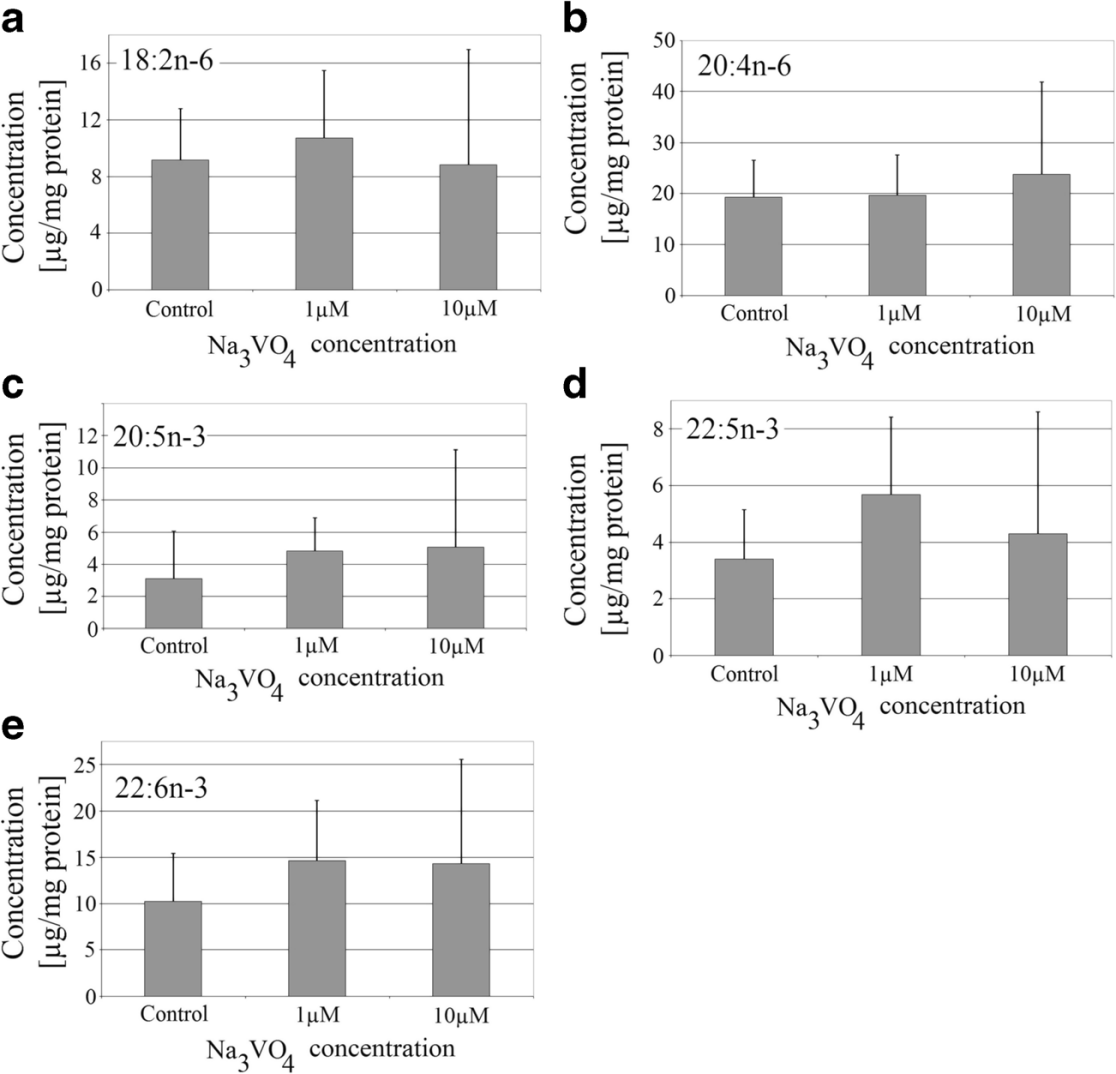
Effect of sodium orthovanadate on PUFA concentration in THP-1 macrophages. The effect of sodium orthovanadate on the amount of **a** linoleic acid, **b** arachidonic acid, **c** eicosapentaenoic acid, **d** docosapentaenoic acid, and **e** docosahexaenoic acid. PMA-activated macrophages of the THP-1 cell line were cultured at two concentrations of sodium orthovanadate. After 48 h of incubation, the cells were scraped and analyzed using a gas chromatograph. Data represent means ± SD for six independent experiments

Sodium orthovanadate at the applied concentrations did not change the amount of polyunsaturated fatty acids (PUFA) in THP-1 macrophages (Fig. 3). The concentration of linoleic acid, arachidonic acid, eicosapentaenoic acid (EPA), docosapentaenoic acid (DPA), and docosahexaenoic acid (DHA) did not significantly change relative to the control with PBS.

### Sodium Orthovanadate Increased the Expression of Stearoyl-Coenzyme A Desaturase

**Fig. 4:**
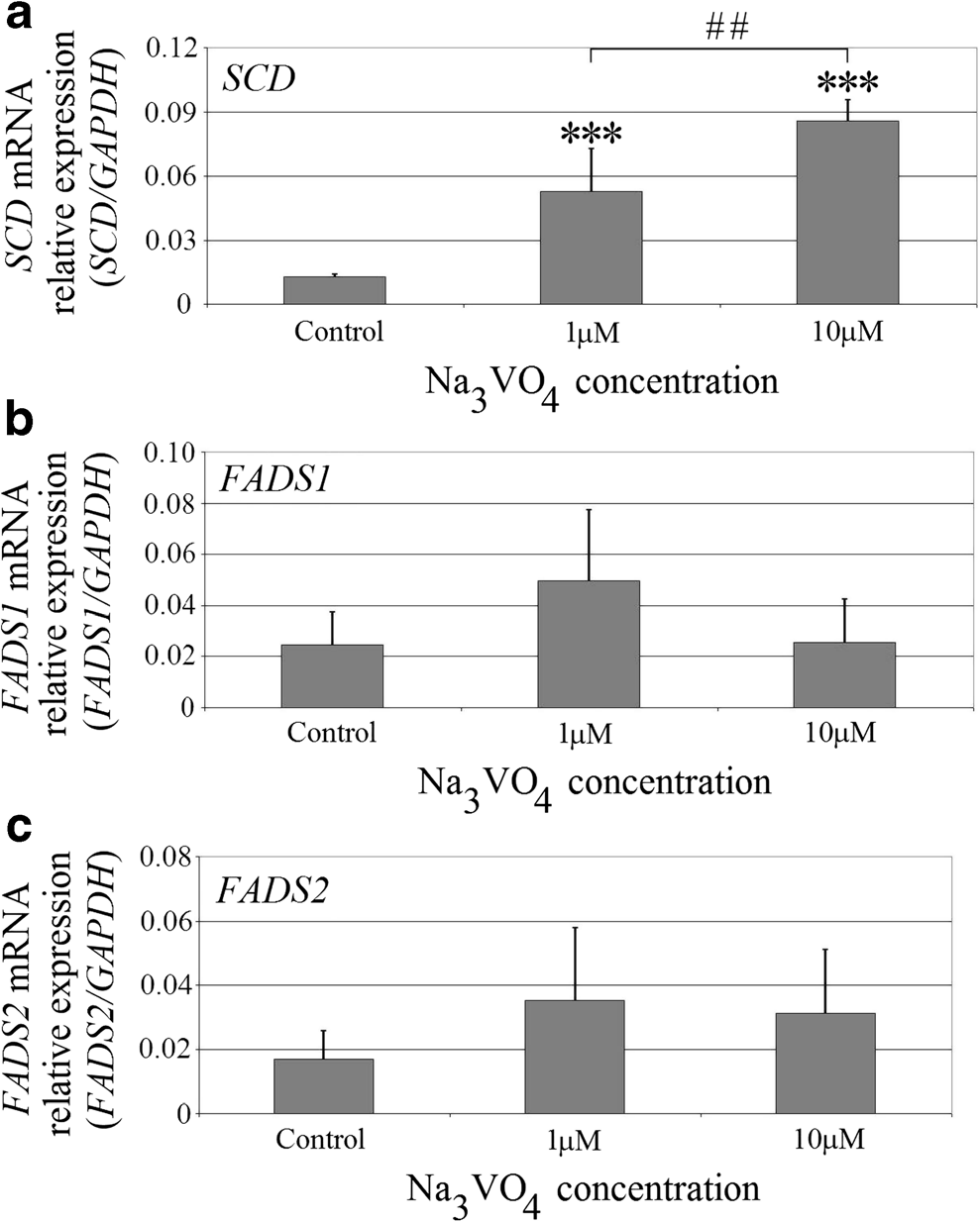
The effect of sodium orthovanadate on the expression of desaturases involved in the conversion of fatty acids. The effect of sodium orthovanadate on the expression of **a***SCD*, **b***FADS1*, and **c***FADS2*. PMA-activated macrophages of the THP-1 cell line were cultured at two concentrations of sodium orthovanadate. After 48 h of incubation, the cells were scraped and analyzed using qRT-PCR. Data represent means ± SD for six independent experiments. Triple asterisks indicate statistically significant difference in the expression of the given gene in macrophages relative to control with PBS, *p* ≤ 0.0001. Double number signs indicate statistically significant difference in expression between two concentrations of sodium orthovanadate, *p* ≤ 0.01

Sodium orthovanadate at the concentrations used increased the *SCD* expression (Fig. 4). At 1 μM and 10 μM, it increased the expression of this gene four times (*p* < 0.0001) and above six times (*p* < 0.0001) relative to control, respectively. At 10 μM, it also significantly increased the *SCD* expression in comparison with 1 μM (*p* = 0.0022). At 1 μM, sodium orthovanadate increased the expression of *FADS1* two times. At all concentrations, it increased *FADS2* expression by two times relative to the control. Nevertheless, the effect on the expression of *FADS1* and *FADS2* was statistically insignificant (*p* > 0.05).

## Discussion

Vanadium compounds are tested as promising drugs against T2DM [14, 15]. In addition to the effects on glucose metabolism, vanadium compounds such as sodium orthovanadate or vanadyl sulfate also reduce plasma cholesterol and LDL [30, 31]. These effects are the result of stimulating glycolysis, glycogen synthesis, and fatty acid synthesis in the liver, muscles, and adipose tissue [28, 31, 49, 58].

In this study, we found that sodium orthovanadate changed the fatty acid composition in THP-1 macrophages, increasing the amount of palmitic and stearic acids, as well as oleic and palmitoleic acids. This is associated with increased expression and activity of fatty acid synthase (FAS) and Δ^9^-desaturase, enzymes responsible for SFA and MUFA biosynthesis. The results obtained in this work confirm numerous scientific reports. In a streptozotocin-induced diabetic rat model which caused deregulation of glucose and lipid metabolism enzymes as well as decreased expression and activity of FAS and acetyl-CoA carboxylase in the liver, an increased expression and activity of these enzymes after exposure to vanadium compounds was demonstrated [28, 31, 58]. This may result in increased plasma glucose uptake by various tissues and its incorporation into fatty acid metabolism, thereby normalizing blood glucose levels. In our study, sodium orthovanadate also increased the expression of *SCD* and hence the activity of Δ^9^-desaturase. The expression of this enzyme is significantly altered by insulin and therefore decreases in diabetes [59]. Therefore, our results are consistent with the previously indicated insulin-enhancing property of vanadium compounds. This confirms the results by Arbo et al. where insulin increased the expression of *SCD* and thus the activity of Δ^9^-desaturase [60].

The present work is the first to show that in addition to the effects on the liver, muscle, and adipose tissue, macrophages under incubation conditions with vanadium compounds may participate in glucose uptake and incorporation into lipid metabolism pathways, thereby contributing to the normalization of blood glucose.

In this work, sodium orthovanadate did not affect the amount of any PUFA in THP-1 macrophages. This was associated with the lack of influence on the expression of *FADS1* and *FADS2*, genes encoding Δ^5^-desaturase and Δ^6^-desaturase, respectively, i.e., enzymes involved in the conversion of α-linolenic acid to EPA, DPA, and DHA and linoleic acid to γ-linolenic acid and arachidonic acid. We were the first to investigate the effect of sodium orthovanadate on the expression of *FADS1* and *FADS2*. The lack of effect on the expression of these enzymes to some extent contradicts the properties of vanadium compounds. In THP-1 macrophages, insulin increases the expression of *FADS1* and *FADS2* and hence the activity of enzymes they encode, i.e., Δ^5^-desaturase and Δ^6^-desaturase [60]. In streptozotocin-induced diabetic rats, disorders in the action of insulin resulted in the reduced expression of Δ^6^-desaturase in the liver [61]. However, a study by Mašek et al. showed that the expression of *FADS2* does not change in diabetic rat liver [59]. The reason for these results may be the very properties of vanadium compounds. In particular, vanadium compounds affect metabolism via insulin-like growth factor 1 receptor (IGF-1R) [62]. Therefore, they have a more inductive effect on the proliferation of cells than insulin. Thanks to this action, vanadium compounds are more effective in increasing the expression of *SCD*, an enzyme involved in the proliferation of cells [63]. Vanadium compounds to a lesser extent affect the expression of *FADS1* and *FADS2* than insulin, which has a greater effect on metabolism. Another significant factor was the concentration of sodium orthovanadate, since the expression of *FADS1* and *FADS2* was affected at the higher concentration used (10 μM).

The results of our work show that sodium orthovanadate can affect the mechanisms involved in the development of atherosclerosis. Increased *SCD* expression and increased Δ^9^-desaturase activity protect macrophages from the proinflammatory action of SFA [64–66]. Among other things, it reduces the activation of NLRP3 inflammasome and NF-κB and thus inflammatory reactions that are important in the development of atherosclerosis. Also, the increased expression and activity of this enzyme causes the efflux of cholesterol from macrophages [67]. These processes protect against the development of atherosclerosis.

On the other hand, in the same model of THP-1 macrophages, sodium orthovanadate increased the synthesis of prostaglandin E_2_ (PGE_2_) with arachidonic acid [68]. This may promote the development of atherosclerosis [69]. In addition, the sodium orthovanadate–induced increase in the amount of fatty acids in THP-1 macrophages demonstrated in our work may be not beneficial. Increased accumulation of SFA may cause inflammatory reactions and uptake of oxidized low-density lipoprotein (oxLDL) [66, 70]. Increased accumulation of SFA in macrophages may disturb cholesterol metabolism in them [71] and contribute to the formation of atherosclerosis, where macrophages are an important link. The accumulation of cholesterol esters derived from lipoproteins is followed by the de-esterification of cholesterol and removal of free cholesterol from these cells [72]. Low activity of FAS increases the process of removing free cholesterol from macrophages and thus slows down atherosclerosis [73].

Vanadium compounds generate ROS, which can consequently accelerate the development of atherosclerosis. As has been shown so far, vanadyl, a vanadium compound at the +4 oxidation state, is responsible for this process. It caused the oxidation of plasma lipids in in vitro and in vivo experiments [30, 74]. After entering the cytoplasm, vanadium compounds at the +5 oxidation state (vanadates, including sodium orthovanadate) are reduced by intracellular antioxidants to vanadium compounds at the +4 oxidation state to give ROS [75, 76]. The inorganic vanadium compounds at the +4 oxidation state undergo Fenton reaction to form ROS and vanadate at +5 oxidation state [77, 78]. A cycle is formed in which ROS is constantly generated, compounds that destructively affect various molecules in the cell and cause formation of oxLDL [70, 74, 79].

The compounds with a large number of double bonds are particularly sensitive to oxidation by ROS. An example of such compounds is PUFA. These fatty acids contain many double bonds in one molecule, making them susceptible to oxidation by ROS. Therefore, increasing the amount of PUFA fatty acids in macrophage cells may exacerbate oxidative stress in them [80–82]. In our study, sodium orthovanadate did not increase the amount of PUFA fatty acids in the studied macrophages. Due to the generation of ROS by vanadium compounds, the lack of effect on the amount of PUFA in cells appears to be a positive property of the vanadium compound tested.

Increased expression of SCD by sodium orthovanadate may intensify cancer mechanisms. During its intensive division, a tumor cell synthesizes its components, including fatty acids, hence the increased expression of *SCD* in tumors, e.g., observed in human hepatocellular carcinoma [83], anaplastic thyroid carcinoma [84], breast cancers [85], prostate cancers [85], or lung adenocarcinoma [86]. The greater the *SCD* expression in a tumor, the worse the prognosis. Therefore, Δ^9^-desaturase inhibitors are being investigated as potential anticancer drugs [87, 88]. If vanadium compounds increase the expression of *SCD*, they also increase tumor growth.

In conclusion, sodium orthovanadate changed SFA and MUFA composition in THP-1 macrophages and increased expression of *SCD*. Sodium orthovanadate did not affect the amount of any PUFA. This was associated with the lack of influence on the expression of *FADS1* and *FADS2*.
